# Potential Immunoregulatory Mechanism of Plant Saponins: A Review

**DOI:** 10.3390/molecules29010113

**Published:** 2023-12-23

**Authors:** Liuhong Shen, Hao Luo, Lei Fan, Xinyu Tian, Anguo Tang, Xiaofeng Wu, Ke Dong, Zhetong Su

**Affiliations:** 1 The Key Laboratory of Animal Disease and Human Health of Sichuan Province, The Medical Research Center for Cow Disease, College of Veterinary Medicine, Sichuan Agricultural University, Chengdu 611130, China; 2 Sichuan Yuqiang Herbal Biotechnology Co., Ltd., Chengdu 611130, China; 3 Guangxi Innovates Medical Technology Co., Ltd., Lipu 546600, China

**Keywords:** plant saponins, phytochemicals, immunity, immunomodulators

## Abstract

Saponins are extracted from different parts of plants such as seeds, roots, stems, and leaves and have a variety of biological activities including immunomodulatory, anti-inflammatory effects, and hypoglycemic properties. They demonstrate inherent low immunogenicity and possess the capacity to effectively regulate both the innate and adaptive immune responses. Plant saponins can promote the growth and development of the body’s immune organs through a variety of signaling pathways, regulate the activity of a variety of immune cells, and increase the secretion of immune-related cytokines and antigen-specific antibodies, thereby exerting the role of immune activity. However, the chemical structure of plant saponins determines its certain hemolytic and cytotoxicity. With the development of science and technology, these disadvantages can be avoided or reduced by certain technical means. In recent years, there has been a significant surge in interest surrounding the investigation of plant saponins as immunomodulators. Consequently, the objective of this review is to thoroughly examine the immunomodulatory properties of plant saponins and elucidate their potential mechanisms, with the intention of offering a valuable point of reference for subsequent research and advancement within this domain.

## 1. Introduction

The immune system assumes a crucial function in identifying and eradicating no-self constituents of antigen foreign entities, while also governing and upholding internal environmental equilibrium [1]. Moreover, it is a complex network, mainly comprising immune organs (spleen, thymus) and immune cells (lymphocytes, monocytes, neutrophils, and macrophages) and it produces specific immune substances (antibodies, cytokines, and chemokines) to provide protection and resistance to various infections and diseases [2,3]. The immune response, while characterized by specificity and efficiency, can give rise to a range of diseases, including autoimmune diseases, hypersensitivity reactions, and immunosuppressive disorders, when its equilibrium is disrupted [4,5]. Currently, according to epidemiological data, there is a notable surge in the prevalence of immune diseases, thereby fostering the advancement of immunomodulators [6]. Immunomodulators encompass a category of both synthetic and natural molecules that possess the capacity to regulate the immune system’s innate and adaptive responses [3]. Pentoxifylline, levamisole, thalidomide, and isoprinosine are extensively utilized synthetic immunomodulators that exert potent regulatory effects on the immune system of organisms [7]. Despite the numerous advantages associated with traditional immunomodulators, they also exhibit a plethora of unforeseen adverse reactions and detrimental effects on the human body [8]. The occurrence of severe adverse drug reactions significantly curtails the sustained utilization of these compounds, thereby necessitating the development of novel approaches that surpass the efficacy and safety of conventional immunomodulators. The utilization of natural plant compounds, including saponins, polysaccharides, and brass, along with their active derivatives, holds significant potential in the modulation of immune responses. In contemporary times, the exploration of plant extracts has garnered substantial attention owing to their diverse pharmacological properties, encompassing immune regulation and antioxidant efficacy [9]. As an innovative form of immunomodulator, plant extracts containing saponins are becoming increasingly popular in clinical practice due to their rich sources, low toxicity, and immunomodulatory properties. In recent years, studies have shown that SARS-CoV-2 S1-Fc candidate vaccine and saponin microemulsion adjuvant have produced high titers of S1 (recombinant protein) specific neutralizing antibodies in cynomolgus monkeys [10], and the effectiveness of *Quillaja brasiliensis* saponins has also been confirmed in experimental vaccines against bovine herpesvirus [11], human poliovirus [12], rabies in mice [13], etc.

**Figure 1:**
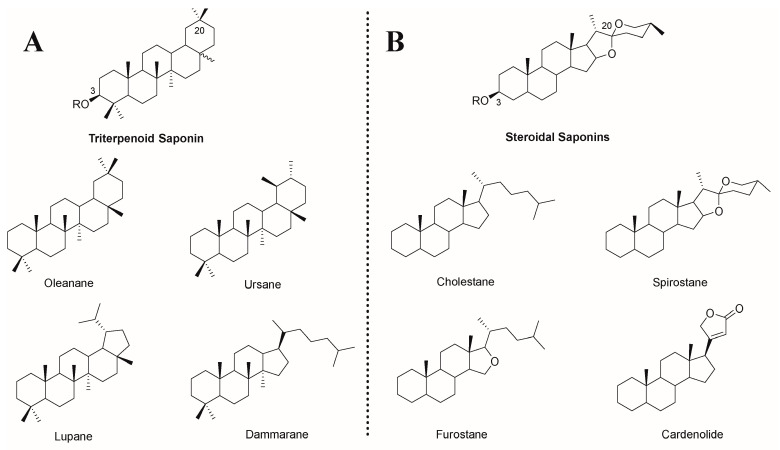
Chemical structures of (**A**) triterpenoid saponins and (**B**) steroidal saponins and their representative sapogenin. R = sugar moiety [14,16].

Saponin is a unique compound extracted from natural plants, and its amphiphilic properties are derived from its structure, which contains an isoprenoid-derived aglycone (a sapogenin) and is linked to one or more sugar chains by ether or ester linkage [14]. Based on its aglycone skeleton, it can be divided into two categories: triterpenoid saponins and steroid saponins [15]. Both of them are extracted from oxidized polymers containing 30 carbon atoms, but the difference is that triterpenoid saponins still retain 30 carbon atoms, while steroidal saponins remove three methyl groups [16]. Among them, triterpenoid saponins are widely distributed in dicotyledons and have four main skeletons: pentacyclic oleanane, ursane, lupane, and tetracyclic dammarane. Steroid saponins are mainly derived from monocotyledons, including four major skeletons of tetracyclic cholestane, hexacyclic spirostane, pentacyclic furostane, and lactone-bearing cardenolide (Figure 1) [14]. According to the number of sugar residues, aglycones are divided into monodesmosidic (one sugar residue), bidesmosidic (two sugar residues), and polydesmosidic saponins (three or more sugar residues) [17].

**Figure 2:**
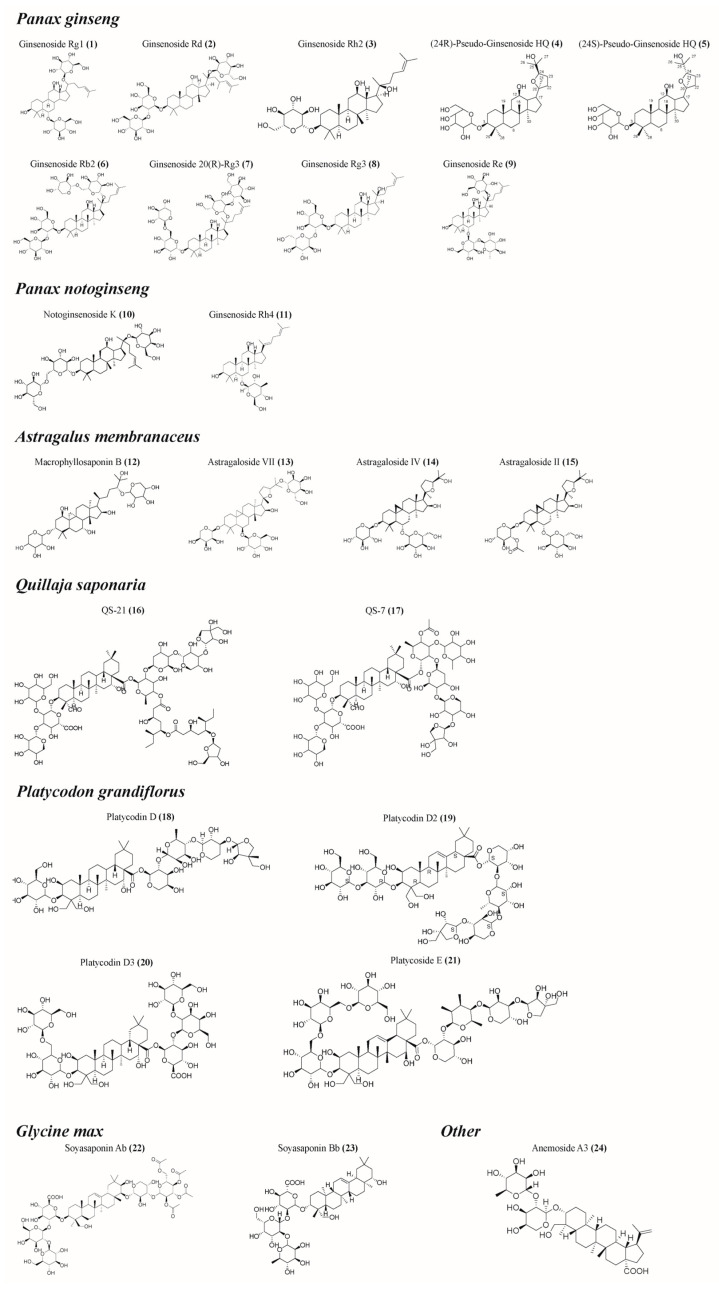
Structural formula of some saponins. (The figure was created using ChemDraw).

Plant saponins have a variety of biological activities, including immune regulation, anti-inflammatory, and hypoglycemic effects [18]. The potential immunomodulatory effects of saponins have attracted great attention since their discovery in 1925 as enhancers of the body’s immune response to diphtheria or tetanus [19]. Since 1964, the saponin *Quillaja saponaria* extracted from the bark of South America has become the main focus of saponin research focusing on immunoregulation activity [20]. However, the availability of *Quillaja saponaria* resources is severely limited, and the potent hemolysis exhibited by *Quillaja* saponins and its isolates, coupled with their instability caused by the presence of ester-bonded structures, has hindered their utilization as immunomodulators. Several plant saponins, including *Panax ginseng* [21,22,23,24,25,26,27], *Panax notoginseng* [28,29,30,31,32,33], and *Astragalus membranaceus* [34,35,36,37,38,39,40], have been shown to have good immunomodulatory activity (Table 1, Figure 2). These saponins are capable of stimulating the mammalian immune system by activating the innate immune response and promoting the generation of cytotoxic T lymphocytes (CTLs) that target exogenous antigens, among other effects [41]. Saponins are widely used alone, and mixed with aluminum salts, liposomes or amphiphilic proteins and lipids to form detergent/lipid/saponin complexes, known as immune-stimulating complexes (ISCOMs) [42]. The majority of research conducted on plant saponins has primarily concentrated on their immune-enhancing properties or their effects on model antigens, lacking a comprehensive synthesis of the immunomodulatory activities exhibited by their active compounds. This paper presents a thorough examination of the immunomodulatory properties exhibited by plant saponins in isolation, along with an initial exploration of the underlying mechanisms involved. The aim is to provide theoretical references for the research, development, and utilization of saponin-based immunomodulators. 

This review used a predefined search strategy to incorporate the research results on the immunomodulatory activity of saponins in articles and reviews published up to 2023. Public databases (PubMed and Web of Science) were used for the literature search. The following keywords were used as search terms in the database: plant saponins, phytochemicals, traditional Chinese herbal medicine, natural products, immune regulation, immunomodulators, immunity, vaccine, immune adjuvant, and immune system.

## 2. Saponins Promote the Growth and Development of Immune Organs

The degree of immune organ development serves as a highly perceptible measure of an organism’s immune status. The amplification and specialization of immune cells within these organs contribute to their augmented mass and subsequent maturation. As two important immune organs, the spleen and thymus state of development can directly reflect the immune function of the body [67]. Notably, the efficacy of various saponins, such as those derived from ginseng, has been demonstrated in augmenting the mass of immune organs and facilitating their favorable development [22]. Additionally, (24R)-pseudo-ginsenoside HQ **(4)**, (24S)-pseudo-ginsenoside HQ **(5)**, and ginsenoside Rb2 **(6)** have been found to restore immune organ function in individuals with compromised immune systems [21,68]. The presence of saponins such as ginsenoside Rg1 **(1)** in the spleen stimulates the release of Th1 and Th2 cytokines by T lymphocytes [21,24] and macrophages [69] in the white marrow [22,34,38,39], thereby producing an immunostimulatory impact. In addition, immunostimulants containing soyasaponin Ab **(22)** and Bb **(23)** can effectively improve the immune status by stimulating the expression of spleen nuclear transcription factor κB (NF-κB), transforming growth factor (TGF-β), and interferon-γ (IFN-γ) [70]. To summarize, phyto-saponins can induce the aforementioned immunostimulatory effects on immune organs, consequently augmenting the overall immune response of the organism.

## 3. Saponins Enhance Immune Cell Activity

The immune system depends on a diverse range of immune cells to carry out its functions. Research has shown that saponins can augment the phagocytic activity of macrophages and the cytotoxicity of natural killer (NK) cells, thereby modulating the innate immune response. Furthermore, saponins can enhance the antigen-presenting capacity of dendritic cells (DCs), activate various subsets of T lymphocytes (such as Th1, Th2, and CTL), induce B lymphocytes to differentiate into plasma cells, and stimulate the production of specific antibodies; these effects collectively contribute to the reinforcement of the adaptive immune response.

### 3.1. Enhancement of Macrophage Activity by Saponins

Macrophages constitute the primary cellular cohort accountable for phagocytosis within the innate immune system, possessing the specialized capacity to identify, internalize, and eradicate a diverse array of bacteria, viruses, and various foreign particles that pose potential harm to the organism [71]. They can participate in phagocytosis, antigen processing, and antigen presentation to lymphocytes, thereby stimulating the production of antigen-specific antibodies and related cytokines [72]. On the contrary, the production of antibodies and cytokines also enhances the chemotaxis and phagocytosis of macrophages [73]. Hence, the process of macrophage phagocytosis exhibits a direct correlation with the efficacy of the body’s immune response. *Panax notoginseng* saponins have been found to enhance the phagocytosis rate of monocytes-macrophages in the presence of immunosuppression, thereby mitigating nonspecific immune injury [33]. Furthermore, astragalosides exert a potent influence on the phagocytic activity of macrophages towards mycobacterium tuberculosis, as well as the stimulation of macrophage secretion of IL-lβ, IL-6, and TNF-α [35]. Following activation, macrophages can be classified into two subtypes: pro-inflammatory macrophages (M1) and anti-inflammatory macrophages (M2) [74]. Anemoside A3 **(24)** can induce the polarization of macrophages towards the M1 phenotype, resulting in an upregulation of their expression of major histocompatibility complex II (MHC II), and release pro-inflammatory cytokines, including IL-6 and tumor necrosis factor-α (TNF-α), to bolster the immune response against pathogenic microorganisms [75,76]. Consequently, saponins could promote the biological activity of macrophages, which in turn effectively enhances the immune response.

### 3.2. Upregulation of NK Cell Killing Ability by Saponins 

NK cells are cytotoxic to virally infected and cancerous cells, as well as modulate other immune cells [77], and studies have shown that platycodin D **(18)** and total saponin from the stem bark of *Albizia julibrissin* effectively upregulate NK cell killing capacity [51,52,53,63]. Whereas NK cell killing capacity depends on the balance of target cell activation and inhibitory receptor expression, and stimulatory and inhibitory ligand expression [78], ginsenoside 20(R)-Rg3 **(7)** can increase the expression of natural cytotoxic receptors including NKp30, NKp44, and NKp46 to enhance NK cell killing capacity [79]. In addition, ginsenoside Rg3 **(8)** upregulates the expression of perforin and granzyme B as well as cytolytic molecules in NK cells to promote their cytolytic activity [79]; perforin and granzyme B are key effector molecules in the NK cell-mediated killing pathway, whereby perforin disrupts the outer membrane of the target cell, allowing the granzyme B to be released into the cytoplasm of target cells, triggering an enzymatic chain reaction leading to target cell death [80]. In conclusion, saponins can upregulate the killing ability of NK cells to enhance the strength of the innate immune response of the body.

### 3.3. Promotion of Dendritic Cell Maturation by Saponins 

DCs play a pivotal role as antigen-presenting cells (APCs) within the immune system, facilitating the efficient processing and presentation of exogenous antigens [81]. In peripheral tissues, immature DCs (iDCs) exhibit a heightened capability to phagocytose and process antigens, effectively capturing them in response to various stimuli, including inflammation or infection. Immature dendritic cells (iDCs) can express pattern recognition receptors (PRRs), including Toll-like receptors (TLRs), and undergo a multifaceted process of differentiation into mature dendritic cells (mDCs) [82]. The co-administration of astragaloside VII **(13)** with LPS resulted in a significant increase in IL-12 secretion by dendritic cells, indicating their maturation [34]. Furthermore, the liposomal preparation of QS-21 **(16)** with a TLR-4 agonist demonstrated enhanced expression of MHC II and CD86 in dendritic cells derived from human monocytes [83]. These findings suggest that saponins can directly promote the maturation of DCs, thereby enhancing the body’s immune response to diseases such as tumors or infections. Moreover, distinct activation pathways are present in various subsets of dendritic cells (DCs) that consume plant saponins. A recent investigation discovered that the PERK pathway was specifically upregulated in mouse CD11b+ MHCII+ DCs when exposed to immunomodulators containing saponins [84], but the precise mechanism remains unknown. However, QS-21 **(16)** can be internalized by DCs in a cholesterol-dependent manner and accumulated in lysosomes, exacerbating lysosomal instability and increasing cathepsin B activity to promote antigen translocation and direct activation in DCs [83]. Additionally, astragaloside VII **(13)** can augment the expression of chemokine receptors, facilitate the conveyance of exogenous antigens to CD8+ T cells via MHC I molecules on DCs [34], and intensify the migration of DCs. In summary, these mechanisms of plant saponins on DCs help to enhance the processing and presentation of antigens to enhance the body’s immune response.

### 3.4. Activation of T and B Lymphocytes by Saponins 

Lymphocytes, being crucial immune cells within the immune system, undergo proliferation and differentiation after their activation, with the extent of their proliferation and activation being capable of reflecting the body’s level of immunity [85]. T lymphocytes play a crucial role in cellular immunity, and the activation of CD4+ Th cells by astragaloside VII **(13)** leads to the production of various cytokines [34]. This activation also facilitates the proliferation and activation of other immune cells, thereby aiding in the development of an effective and protective immune response [21,34]. Additionally, QS-21 **(16)** and QS-7 **(17)** activate CD8+ CTLs, enhancing their ability to destroy target cells [43,44,49,50]. B lymphocytes can undergo differentiation into effector B cells in response to immunogenic substances and a cascade of cytokines released by Th cells and APCs. These effector B cells are responsible for the synthesis and secretion of immunoglobulins, actively contributing to the humoral immune response [86]. Saponin, including *Panax notoginseng* saponins, can stimulate mature B lymphocytes to generate antigen-specific antibodies via NF-κB and other signaling pathways. Moreover, it enhances the longevity of memory B cells and specific plasma cells within the bone marrow, thereby enhancing the humoral immune response of the organism [87,88]. CD4 T cells that are stimulated in the presence of plant saponins exhibit elevated levels of IL-21, a crucial cytokine for T follicular helper cells. This cytokine plays a significant role in promoting B lymphocyte proliferation, isotype switching, and differentiation [89]. Conversely, the substantial early expression of IFN-α and IL-6 induced in the draining lymph nodes may contribute to the proliferation and differentiation of B lymphocytes [90,91]. Furthermore, a separate study has demonstrated that astragaloside IV **(14)** can modulate the balance between Th17 and Treg cells, thereby effectively preventing and alleviating immune-related damage within the body [92]. In vitro assays demonstrated that *Albizia julibrissin* saponins, *Achyranthes bidentata* saponins, *Asparagus adscendens* saponins, and the pods of *Acacia concinna* saponins induced a gradual transformation of lymphocytes into lymphoblastoid cells and enhanced lymphocyte sensitivity to pertinent stimuli and cellular functionality [63,64,65,66]. In summary, saponins have the potential to augment the expression of diverse cytokines, thereby promoting the proliferation and activation of T and B lymphocytes and optimizing the distribution of various lymphocyte subpopulations. Consequently, this mechanism can regulate the immune status of the organism and enhance its capacity to mount an immune response.

## 4. Saponins Upregulate the Expression of Immunomodulatory Molecules

Saponins can stimulate the release of cytokines and chemokines during the immune response, regulate immune cell recruitment and intracellular signaling mechanisms, facilitate the proliferation and differentiation of T lymphocytes into CD4+ Th cells for involvement in diverse immune responses, and enhance the production of antigen-specific antibodies by B lymphocytes, thereby augmenting the humoral immune response of the organism.

### 4.1. Regulation of Cytokine and Chemokine Expression by Saponins

The activation of T lymphocytes is contingent upon three distinct signals that occur between the antigen-presenting cell (APC) and the T lymphocyte. These signals encompass the stimulation of the T cell receptors (TCRs) and major histocompatibility complex II (MHC II) on the APC by the antigen, the direct impact on the T lymphocyte through co-stimulatory molecules present on the APC, and the regulation exerted by cytokines secreted by the APC [93]. Upon activation, initial T lymphocytes can undergo differentiation into various effector T cells, such as Th1, Th2, and Th17 [94]. Ginseng saponin immunomodulators have been observed to stimulate the production of cytokines, including TNF-α, by Th1 cells. This stimulation enhances the organism’s capacity to eradicate cancer cells, inhibit viral replication in macrophages, and augment antimicrobial efficacy, among other effects [22,23,27]. In addition, platycodin D **(18)** and platycodin D2 **(19)** can promote the secretion of cytokines by Th2 cells, namely, IL-4, IL-5, IL-10, and IL-13, thereby stimulating B lymphocyte proliferation and subsequent transformation into plasma cells, and promoting antigen-specific antibody production. IL-4 and IL-5 also cause eosinophils and mast cells to degranulate, making the parasite fragile and enhancing the body’s defense against parasitic infections [52,53,54,55,56,58]. Astragaloside IV **(14)** exhibits a specific activating influence on Th17 cells, which assume a pivotal role in the activation and recruitment of neutrophils to immune sites, and are capable of secreting IL-6, IL-17, and IL-22 to combat extracellular pathogens [92,95]. The chemokine family plays a crucial role in facilitating tissue-specific migration of immune cells, enabling their movement in response to a concentration gradient of chemokines, thereby promoting a heightened immune response [96]. It has been observed that *Albizia julibrissin* saponins can enhance the expression of chemokines in the thymus after immunization, including fractalkine and macrophage colony-stimulating factor (M-CSF), recruit DCs at the immune site and activate macrophages, induce monocytes to differentiate into DCs and macrophages, mediate the migration and adhesion of various leukocytes, stimulate granulocytes, and enhance the body’s antigen-specific immune response [63]. In summary, the utilization of saponin demonstrates its ability to modulate the concentrations of diverse cytokines and chemokines, thereby achieving a more optimal equilibrium between the Th1/Th2 immune response. Additionally, these immunomodulators facilitate the recruitment of innate immune cells to the site of injection, thereby augmenting the overall immune response.

### 4.2. Promotion of Antibody Secretion by Saponins

Antibodies, referred to as immunoglobulins, are produced by B lymphocytes in reaction to external antigenic stimulation [97]. These antibodies can be categorized into different subtypes, such as IgG, IgA, and IgM. It is worth noting that the levels of the IgG1 subtype are correlated with Th2 immune response, whereas the levels of IgG2a, IgG2b, and IgG3 subtypes are associated with Th1 immune response [98]. Some of the saponins, like astragaloside VII **(13)**, IV **(14)**, and II **(15)** can induce cytokine secretion, which in turn regulates the expression of germline gene transcripts (GLTs) and the secretion of corresponding immunoglobulins in B cells. Specifically, they promote the secretion of Th1 cytokines (such as IL-2 and IFN-γ) to regulate the production of Th1-dependent antibodies. Additionally, they upregulate Th2 cytokines (such as IL-4 and IL-10) to enhance the production of Th2-dependent antibodies and improve the body’s immune responses, including antitoxin, antibacterial, antiviral, and allergy modulation [35,36,37]. Ginseng stem leaf saponins can also improve the activity of intestinal intraepithelial lymphocytes in the lamina propria of duodenum, jejunum, and ileum and induce the conversion of B lymphocytes into IgA plasmablasts, further maturing into IgA plasma cells and secreting IgA, thereby providing an effective mucosal immune response [99]. While IgM is detectable before antigen exposure and serves as an intrinsic defense mechanism preceding adaptive immunity, ginseng stem leaf saponins possess the capability to augment the production and secretion of IgM within the organism to a certain extent [100,101]. In summary, the utilization of saponin immunomodulators has been found to enhance humoral immune responses through the augmentation of immunoglobulin levels.

## 5. Saponins Modulate Immune-Related Signaling Pathways

Saponins can induce immune cell activation and cytokine release through various immune-related signaling pathways such as TRLs, NF-κB/mitogen-activated protein kinase (MAPK), and hippo-YAP, thereby initiating innate immune responses and promoting antigen-specific immune responses.

**Figure 3:**
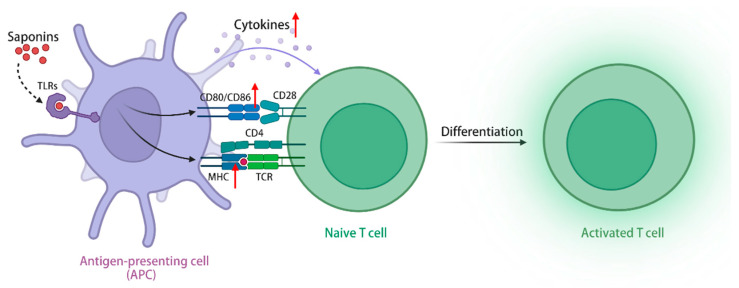
Saponins activate the TLRs signaling pathway through TLRs, upregulate class I and II MHC molecules in APCs, and promote antigen capture, processing, and presentation. (The figure was created using BioRender).

TLRs are a class of transmembrane receptors responsible for recognizing exogenous microorganisms, including bacteria and viruses, and initiating immune responses, and the TLRs signaling pathway plays a crucial role in enabling immune cells to identify pathogens and release immunomodulatory factors [102]. In addition to exogenous pathogens serving as ligands, TLRs exhibit the recognition of endogenous plant-derived molecules, including saponins, polysaccharides, flavonoids, etc., thereby initiating subsequent signaling cascades [103]. Upon activation of the TLRs signaling pathway, ginseng stem leaf saponins can augment the migratory capabilities of immune cells, increase the expression of MHC class I and II molecules in APCs, and facilitate the capture, processing, and presentation of antigens [104,105]. Furthermore, the stimulation of TLRs signaling pathway by ginsenoside Rg1 **(1)** and Re **(9)** has the potential to elicit an immune-protective cytokine response, enhance the expression of costimulatory molecules CD40, CD80, CD86, and CD70 in antigen-presenting cells, and generate immune-related cytokines (Th1 and Th2), including IL-2, IL-6, and IL-12 [106]. The simultaneous administration of QS-21 **(16)** and TLR agonists demonstrates a synergistic effect, resulting in a significant increase in the levels of antigen-specific antibodies. Only the combined use of both components can induce a remarkable expansion in the population of antigen-specific CD4+ Th1 cells, thereby promoting a cellular immune response [107]. In conclusion, saponins can activate APCs via the signaling pathway of TLRs, leading to enhanced antigen processing, increased secretion of immune-related cytokines, improved stimulation of T and B lymphocytes, and heightened cellular and humoral immune responses, thereby demonstrating their immunomodulatory effect. Furthermore, when combined with TLRs activators, saponins exhibit a substantial enhancement in their immunomodulatory activity (Figure 3).

**Figure 4:**
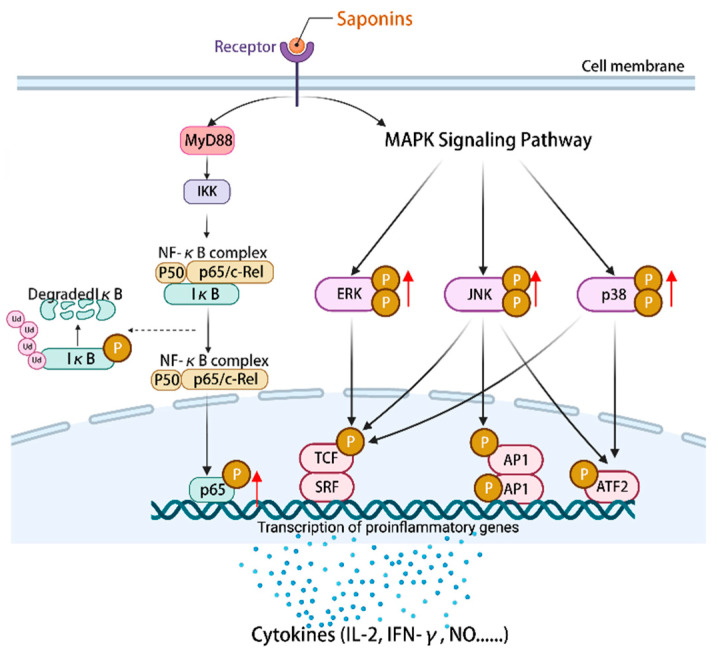
Activation of the NF-κB/MAPK signaling pathway by saponins regulates the expression of a series of immune-related cytokines, NO, surface-stimulating factors, and mRNAs and/or proteins of the cell cycle. (The figure was created using BioRender).

NF-κB is an important component of the immune system [108]. MAPK can regulate gene expression, immune response cell proliferation, and other cellular processes [109]. The mechanism by which astragalosides exert their effects involves the regulation of downstream immune factors within the NF-κB/MAPK signaling pathway, including IL-2, IFN-γ, and TNF-α [110]. Astragaloside IV **(14)** can stimulate an increase in the expression of p-p65/p65 proteins and facilitate the phosphorylation of p38, ERK, and JNK. This activation of the NF-κB/MAPK signaling pathway subsequently governs the modulation of pro-inflammatory and anti-inflammatory cytokines, nitric oxide (NO), surface stimulating factors, as well as the mRNA and protein expression associated with the cell cycle [111]. Ultimately, these mechanisms contribute to the enhancement of the body’s immune function. Furthermore, the study revealed that astragaloside IV **(14)** exhibited a partial inhibitory effect on the differentiation of macrophages into M2 phenotype by modulating the MAPK signaling pathway, consequently impeding cellular invasion, migration, and angiogenesis [112]. Additionally, astragaloside IV **(14)** exerted anti-tumor immunomodulatory effects by regulating the levels of cyclin D1, CDK4, and CDK6, stimulating the expression of costimulatory molecules including CD40 and CD86, and inducing cell cycle arrest in the G2/M phase [111]. To summarize, the activation of the NF-κB/MAPK signaling pathway by plant saponins can effectively regulate the expression of immune molecules downstream, thereby demonstrating promising anti-tumor properties and the potential to enhance immune responses (Figure 4).

**Figure 5:**
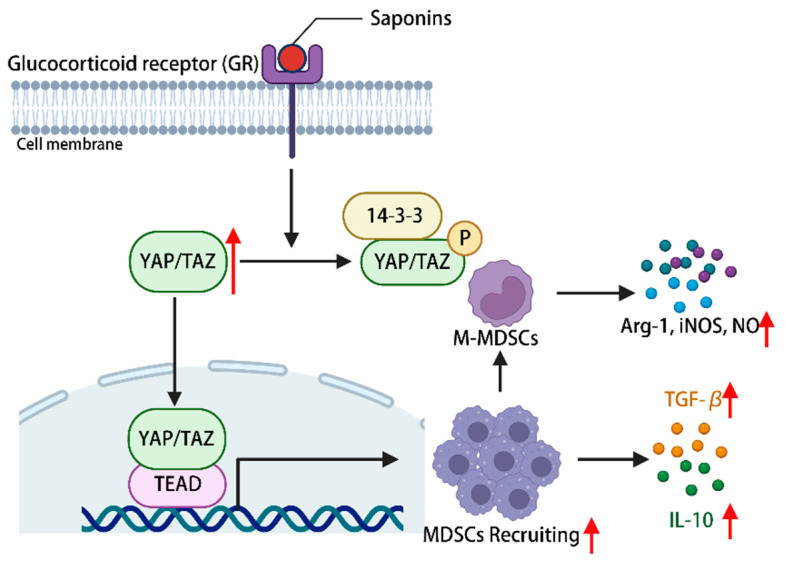
Phytosaponins stimulate the glucocorticoid receptor and activate the hippo-YAP/TAZ signaling pathway, leading to the recruitment of MDSCs as well as promoting their proliferation and differentiation into M-MDSCs, during which a series of cytokines are produced to regulate the immune response of the body. (The figure was created using BioRender).

The constituents of the hippo-YAP/TAZ signaling pathway hold significance in immune regulation, with YAP being deemed essential for the proper functioning of Treg cells [113]. The promotion of myeloid-derived suppressor cells (MDSCs) recruitment is facilitated by the activation of the hippo-YAP signaling pathway [114]. Ginsenoside can activate YAP/TAZ-TEAD via glucocorticoid receptors, consequently facilitating the proliferation and differentiation of MDSCs into fully developed granulocytes, DCs, and macrophages. This activation also leads to an upregulation in the expression of IL-10 and TGF-β, allowing for the infiltration of corresponding tissues and organs, ultimately exerting a normal immune response [115]. Additionally, it has the potential to induce the differentiation of myeloid-derived suppressor cells (MDSCs) into mononuclear MDSCs, thereby augmenting the secretion of arginase-1, inducible nitric oxide synthase (iNOS), and nitric oxide. This mechanism aims to restrain excessive immune activation and maintain immune homeostasis within the body [115]. In conclusion, the activation of the hippo-YAP signaling pathway by plant saponins has been found to regulate the immune microenvironment of MDSCs, thereby demonstrating immune regulatory properties (Figure 5).

In conclusion, plant saponins possess immunomodulatory activity and can regulate the immune function of the body through different pathways. In addition, to a certain extent, plant saponins have the potential to become innovative immunomodulators.

## 6. Limitations of Saponins as Potential Immunomodulators

Disadvantages such as varying degrees of hemolysis, cytotoxicity, poor solubility, and tissue irritation greatly limit the clinical use of saponins. Saponins tend to bind to the cholesterol (Cho) present in the erythrocyte membrane, resulting in the formation of insoluble complexes. Consequently, the altered osmolality eventually triggers erythrocyte swelling and rupture, ultimately leading to hemolysis [116,117]. In addition, the chemical structure of saponins makes its solubility low, which may cause pain at the injection site. Therefore, in the use of saponin preparations, it is best to choose oral administration to avoid intravenous and intramuscular injection.

At present, the focus of the pharmaceutical field is to use intermolecular forces including hydrogen bonds, hydrophobic forces, and salt bonds to convert drugs, especially low-solubility drugs into nano-preparations to avoid some side effects of drugs [118,119]. Nanosizing, a technique for formulating drug powders into particles of nano-scale, is recognized for its ability to enhance drug absorption and enable the intravenous administration of insoluble drugs [120]. Nanopharmaceuticals offer a solution to the issue of drug solubility, while also reducing toxicity and exhibiting a high drug loading capacity [121]. Although this research is still in its infancy, partial laboratory and clinical results have been achieved to date. Through the encapsulation of *Q. brasiliensis* QB-80 saponin (QB-80) within lipids, the creation of a nano-adjuvant (IMXQB-80) was achieved. Toxicity assessments revealed that IMXQB-80 exhibited a notable reduction in cytotoxicity. Furthermore, the nano-adjuvant IMXQB-80 demonstrated comparable efficacy to QB-80 in eliciting immune responses, albeit with a four-fold decrease in the required saponin dosage for achieving equipotent stimulation [122]. The utilization of a nanoparticle encapsulating panax ginseng saponin R1 (NGR1) facilitates the accurate and targeted transportation of NGR1 to various organs, employing a non-invasive approach. This technique exhibits enhanced functionality and angiogenesis within the intended organ, while concurrently mitigating apoptosis [123]. In comparison to other frequently employed immunomodulators, saponin immunomodulators possess both merits and demerits. To enhance the benefits or mitigate the drawbacks, diverse approaches such as nano-preparations and alternative immunomodulator delivery systems can be employed, thereby fostering a safer and more extensive clinical utilization of saponins.

## 7. Conclusions

Phytosaponins can promote the growth and maturation of immune organs, regulate the function of a variety of immune cells, increase the production of immune-related cytokines and antigen-specific antibodies, etc., through a variety of signaling pathways, thus exerting immunomodulatory effects. Due to the special chemical structure of plant saponins, while exerting its immune-enhancing effect, it also possesses certain hemolytic effects and cytotoxicity, which limits its application. However, these drawbacks can be circumvented to a certain extent through different preparation forms and delivery systems. Therefore, saponins have great potential in the development and application of immunomodulators.

## Figures and Tables

**Table 1:** Immunomodulatory activity of some saponins.

Saponin Sources	Saponin Name	Immunoreaction
*Panax ginseng*	Ginsenoside Rg1 **(1)** Ginsenoside Rd **(2)** Ginsenoside Rh2 **(3)** (24R)-Pseudo-Ginsenoside HQ **(4)** (24S)-Pseudo-Ginsenoside HQ **(5)** Ginsenoside Rb2 **(6)** Ginsenoside 20(R)-Rg3 **(7)** Ginsenoside Rg3 **(8)** Ginsenoside Re **(9)**	Facilitating the upregulation of Th1 (IFN-γ, IL-2, T-bet, etc.) and Th2 (IL-4, IL-6, GATA3, etc.) cytokines and transcription factors [22,23,27]; Enhancing the levels of antigen-specific antibodies enhances the body’s immune defense against antigens [23,24,25]; Stimulating lymphocyte proliferation [21,24,25,26]; Preventing atrophy of immune organs [21]; Upregulating the CD4+/CD8+ T cell ratio [21].
*Panax notoginseng*	Notoginsenoside K **(10)** Ginsenoside Rh4 **(11)**	Enhancing the levels of antigen-specific antibodies enhances the body’s immune defense against antigens [29,30,31,32,33]; Ameliorating antigen-induced oxidative stress in immune cells [28]; Improvement of monocyte and macrophage carbon scavenging [33]; Stimulating lymphocyte proliferation [30,31,32,33]; Promoting the secretion of cytokines such as TNF-α and IL-2 [33].
*Astragalus membranaceus*	Macrophyllosaponin B **(12)** Astragaloside VII **(13)** Astragaloside IV **(14)** Astragaloside II **(15)**	Promoting the maturation and activation of APCs such as macrophages or dendritic cells [34,35]; Enhancing the levels of antigen-specific antibodies enhances the body’s immune defense against antigens [35,36,37]; Stimulating lymphocyte proliferation [38,39]; Stimulating the release of cytokines (IL-1β, IL-2, IL-4, IFN-γ, TNF-α, etc.) from immune cells to balance the Th1/Th2 of the organism [38,39]; Activation of CD4+ and CD8+ T cells [34]; Inhibition of TLR2 activator-induced reduction in CXCR4 expression and neutrophil migration improves the body’s antimicrobial immunity [40].
*Quillaja saponaria*	QS-21 **(16)** QS-7 **(17)**	Enhancing the levels of antigen-specific antibodies enhances the body’s immune defense against antigens [43,44,45,46,47,48]; Stimulating the production of CTLs [43,44,49,50]; Promoting the secretion of relevant cytokines and inducing a balanced Th1/Th2 response [43,44,49,50].
*Platycodon grandiflorus*	Platycodin D **(18)** Platycodin D2 **(19)** Platycodin D3 **(20)** Platycoside E **(21)**	Enhancing the levels of antigen-specific antibodies enhances the body’s immune defense against antigens [51,52,53,54,55,56,57]; Stimulating the proliferation of immune cells such as lymphocytes and monocytes [51,52,53,54,55,56,57,58]; Increasing the killing activity of NK cells and CTL [51,52,53]; Facilitating the upregulation of Th1 (IFN-γ, IL-2, T-bet, etc.) and Th2 (IL-4, IL-6, GATA3, etc.) cytokines and transcription factors for a better balance of Th1/Th2 immune responses [52,53,54,55,56,58].
*Glycine max*	Soyasaponin Ab **(22)** Soyasaponin Bb **(23)**	Enhancing the levels of antigen-specific antibodies enhances the body’s immune defense against antigens [59,60]; Activating TRL4/NF-κB signaling [59]; Stimulating lymphocyte proliferation [61]; Stimulation of CD4+ and CD8+ T cells to bind to the antigen [62]; Promoting the secretion of cytokines such as TNF-α and IFN-γ [59,61]; Promoting elevated blood leukocyte, lymphocyte, and monocyte counts within the physiologic range [61].
Other	Anemoside A3 **(24)** *Achyranthes bidentata* saponins *Albizia julibrissin* saponins *Asparagus adscendens* saponins *Momordica charantia* saponins *Acacia concinna* saponins	Enhancing the levels of antigen-specific antibodies enhances the body’s immune defense against antigens [63,64,65,66]; Stimulation of lymphocyte proliferation [63,64,65,66]; Enhancing natural killer (NK) cell killing activity [63]; Promoting the secretion of cytokines such as IL-12 [65]; Increasing CD3/CD19 expression in spleen and lymph nodes [65]; Induction of injection site cytokines (IL-12p40, IL-12p40/p70, IFN-γ, IL-1β, IL-3, IL-6, IL-9, IL-10, IL-13, TNF-α, sTNFR I, and sTNFR III) and chemokines (eotaxin, I-TAC, MIG, MIP-1α, RA, N T-E S, TECK, fractalkine, fasL, M-CSF, and GM-CSF) are expressed to promote immune cell recruitment at the injection site [63].

## Data Availability

Not applicable.
